# Is vitamin D status relevant to psoriasis and psoriatic arthritis? A retrospective cross-sectional study

**DOI:** 10.1590/1516-3180.2022.0216.R1.01072022

**Published:** 2022-09-06

**Authors:** Shirley Braga Lima Gamonal, Aloisio Carlos Couri Gamonal, Nathália Couri Vieira Marques, Marcos Antônio Fernandes Brandão, Nádia Rezende Barbosa Raposo

**Affiliations:** I MD, MSc, PhD. Researcher, Physician and Professor, Núcleo de Pesquisa em Dermatologia (NUPEDE), Faculty of Medicine, Universidade Federal de Juiz de Fora (UFJF), Juiz de Fora (MG), Brazil; Researcher, Núcleo de Pesquisa e Inovação em Ciências da Saúde (NUPICS), Faculty of Pharmacy, Universidade Federal de Juiz de Fora (UFJF), Juiz de Fora (MG), Brazil.; II MD, MSc, PhD. Physician and Professor, Núcleo de Pesquisa em Dermatologia (NUPEDE), Faculty of Medicine, Universidade Federal de Juiz de Fora (UFJF), Juiz de Fora (MG), Brazil.; III Undergraduate Student, Medicine, Núcleo de Pesquisa em Dermatologia (NUPEDE), Faculty of Medicine, Universidade Federal de Juiz de Fora (UFJF), Juiz de Fora (MG), Brazil.; IV PhD. Pharmacist and Professor, Núcleo de Pesquisa e Inovação em Ciências da Saúde (NUPICS), Faculty of Pharmacy, Universidade Federal de Juiz de Fora (UFJF), Juiz de Fora (MG), Brazil.; V MSc, PhD. Pharmacist and Professor, Núcleo de Pesquisa e Inovação em Ciências da Saúde (NUPICS), Faculty of Pharmacy, Universidade Federal de Juiz de Fora (UFJF), Juiz de Fora (MG), Brazil.

**Keywords:** Psoriasis, Arthritis, psoriatic, Vitamin D, Retrospective studies, Prevalence, Psoriatic arthritis, 25-Hydroxyvitamin D, Psoriasis area and severity index, Classification criteria for psoriatic arthritis, Retrospective cross-sectional study

## Abstract

**BACKGROUND:**

Psoriasis is a systemic, immune-mediated disease characterized by inflammatory manifestations in the skin and joints. Vitamin D deficiency is currently considered a pandemic and is associated with comorbidities including psoriasis and psoriatic arthritis (PsA).

**OBJECTIVES:**

To determine the prevalence of hypovitaminosis D [25(OH)D] in patients with plaque psoriasis, with and without PsA, and of independent predictors of serum 25(OH)D levels.

**DESIGN AND SETTING:**

Retrospective cross-sectional study conducted among 300 patients at an outpatient clinic in a university center in Juiz de Fora, Minas Gerais, Brazil.

**METHODS:**

Demographic and clinical data (psoriasis area and severity index [PASI], family history, age at onset, disease duration, and the presence of PsA according to Classification Criteria for Psoriatic Arthritis), skin phototype, and season of the year were reviewed.

**RESULTS:**

Hypovitaminosis D (< 30 ng/mL) was highly prevalent in patients with psoriasis with and without PsA (82.2% and 74.9%, respectively). An inverse correlation between PASI and vitamin D was found (without PsA r = –0.59 and, PsA r = –0.52, P < 0.001), and multivariate regression revealed that hypovitaminosis D was associated with disease severity, season, and phototype. It was confirmed by binary logistic regression between PASI and vitamin D deficiency (< 30 ng/mL), (odds ratio, OR 1.78 CI: –0.20–0.53, P < 0.001).

**CONCLUSION:**

Hypovitaminosis D (< 30 ng/mL) was highly prevalent in psoriatic patients with and without PsA. Season and skin phototype were associated with 25(OH)D levels. An inverse association between PASI and serum 25(OH)D levels was established.

## INTRODUCTION

Psoriasis is a chronic disease with a genetic predisposition. It involves the skin, joints, and immune system,^1^ and is characterized by sustained inflammation with alterations in the proliferation and differentiation of keratinocytes.^2^ The pathogenesis of psoriasis is still not completely understood. However, it is already known that the development of psoriasis plaques is mediated by Th1, Th17, and Th22 cells, with consequent hyperproliferation of keratinocytes.^2^ Vitamin D is considered one of the most important modulators of the immune response, with effects on both innate and adaptive immunity in addition to having antiproliferative actions on keratinocytes.^2,3^ Moreover, beneficial effects of ultraviolet radiation in the treatment of psoriasis reinforce this hypothesis.^2–4^ The role of vitamin D in psoriasis has been studied for over 60 years, since vitamin D analogs, such as calcipotriol, were first used to treat psoriasis. Currently, vitamin D deficiency is considered a worldwide epidemic with multiple implications for human health because of the roles of vitamin D in various physiological systems. Vitamin D deficiency increases the risk of cardiovascular and metabolic diseases, cognitive and affective disorders, and osteoporosis. Chronic inflammation present in patients with psoriasis and psoriatic arthritis could be related to a higher risk of metabolic syndrome and cardiovascular disease in these individuals.^4,5,6,7^

The definition of vitamin D deficiency is still controversial. The Institute of Medicine (IOM) of the National Academy considers vitamin D deficiency 25(OH)D values below 20 ng/mL (or 50 nmol/L) while other societies such as Endocrine Society, National Osteoporosis Foundation, International Osteoporosis Foundation, American Geriatric Society, suggest that the minimum value necessary to reduce the risk of falls and fractures is 30 ng/mL (or 75 nmol/L).^6,7^ The World Health Organization advises serum levels above 30 ng/mL (or 75 nmol/L).^8^ It is believed that the recommended serum levels should be higher in psoriasis and psoriatic arthritis than in the general population, as the scientific literature suggests an association between these two diseases and inadequate levels of vitamin D.^9,10^

However, reports of the relationship among vitamin D, psoriasis, and psoriatic arthritis have come from studies with different methodological approaches and demographically different populations from different geographic regions.

## OBJECTIVE

In this context, this study aimed to determine the prevalence of hypovitaminosis D [25(OH)D] in patients with plaquepsoriasis with and without psoriatic arthritis (PsA) treated at a Psoriasis outpatient clinic. A second aim was to determine independent predictors of serum 25(OH)D levels, such as Fitzpatrick's phototype^11^ and season of the year.

## METHODS

### Sample selection and ethics compliance

We conducted a cross-sectional, comparative, retrospective study that included 300 patients with plaque psoriasis who were treated in psoriasis outpatient clinics of a Dermatology Service between January and December 2016. This study reviewed 350 medical records of patients treated at the psoriasis outpatient clinic and given a standardized medical record that included their serum levels of 25(OH)D. Fifty of the 350 patients were excluded. Patients were excluded due to a lack of accordance with the inclusion and exclusion criteria and missing data in the medical records. As the medical records for this study were obtained from the Dermatology Service of a University Hospital, the data were collected by postgraduate doctors who were also supervised by doctors, and standardized medical records for patients with psoriasis were completed. The inclusion criteria were as follows: patients of both sexes, aged between 18 and 60 years, with clinical and/or histopathological diagnosis of plaque psoriasis, with or without diagnosis of psoriatic arthritis according to the Classification Criteria for Psoriatic Arthritis (CASPAR) criteria.12 The following were excluded: patients with other clinical forms of psoriasis and those who had data missing from the standardized psoriasis medical record, or with severe and decompensated systemic diseases (hepatic, renal, metabolic, or cardiac), thyroid and parathyroid diseases, malignant neoplasms, acquired immunodeficiency syndrome, and pregnant women; patients with diseases with altered intestinal absorption and other autoimmune and photosensitive diseases; patients using oral supplementation of vitamin D, bisphosphonates, systemic corticosteroids, or calcium; patients undergoing treatment by phototherapy or using sunscreens; and patients using topical vitamin D analogs such as calcipotriol. Data collection began after approval of the investigation by our institution's ethics committee (protocol 3.142.153, approved on November 2, 2019, by the Research Ethics Committee of the University Hospital). All the procedures involved in this study were in accordance with the Declaration of Helsinki of 1975, as updated in 2013.

### Clinical, laboratory and radiographic evaluation

Standardized records of patients with psoriasis were used and the following variables were evaluated: sex, age, family history of psoriasis, age at disease onset, duration of the disease, presence of PsA according to CASPAR^12^, and disease severity according to the psoriasis area and severity index (PASI).^13^ Using PASI, the severity of psoriasis was stratified as mild (PASI < 10) or moderate-to-severe (PASI > 10).^13^ Moreover, the patient's phototype and 25(OH)D dosing station were evaluated. The analysis of serum levels of 25(OH)D was performed at the Biochemistry Laboratory of the University Hospital using the chemiluminescence technique (ARCHITECT 25-OH Vitamin D, Abbott Diagnostics, Lake Forest, Illinois, United States) and considered the following parameter definitions: values < 20 ng/mL were considered deficient; ≥ 20 ng/mL and < 30 ng/mL, insufficient; and ≥ 30 ng/mL, sufficient.^14^ Rheumatoid factor and radiographic reports were also reviewed.

### Statistical analyses

A descriptive data analysis was performed. The Shapiro-Wilk test was used to assess the distributions of variables. Student's t-test was used to test the differences in quantitative variables between two groups and these were confirmed by one-way analysis of variance (F test), followed by the Bonferroni post hoc correction. The chi-square test (χ2), or Fisher's exact test when there were less than five data points were used to test for possible differences in the proportions of qualitative variables.

Pearson's coefficient (r) was used to test the correlations between 25(OH)D and continuous variables. To assess independent predictors of vitamin D levels, a multiple linear regression model was developed using vitamin D levels as the outcome and sex, age, phototype, season of vitamin D blood testing, arthritis, family history, age at diagnosis, duration of psoriasis, and disease severity as determinants, and controlling, if necessary, for confounding variables such as sex and age. 

In this context, the presence of arthritis, severity according to PASI, family history, age at diagnosis, evolution time, phototype, and season were used as predictor variables, controlling for sex and age.

In the binary regression, vitamin D was dichotomized as deficient (< 30 ng/mL) or sufficient (≥ 30 ng/mL), and clinical parameters related to psoriasis and psoriatic arthritis were used as predictors of vitamin D levels. The significance level was set at 5% (P < 0.05) for all statistical analyses. Analyses were performed using the R software package for Windows [R Core Team (2019) R, version 3.4.4. (R Foundation for Statistical Computing, Vienna, Austria) and https://www.R-project.org/].

## RESULTS

**Figure 1:**
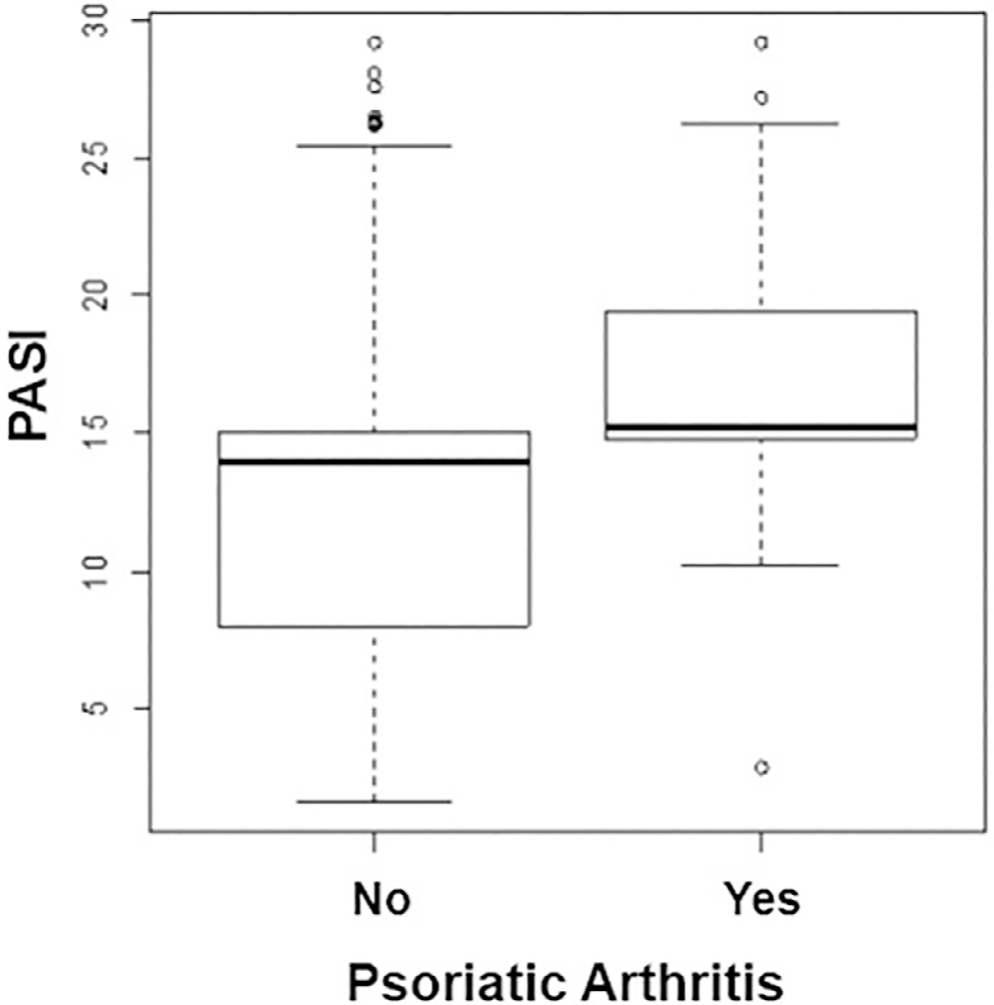
Comparison of the Psoriasis Area and Severity Index (PASI) between psoriasis patients with and without psoriatic arthritis.

The characteristics of patients with plaque psoriasis with and without arthritis are shown in Table 1. Of the 300 patients with plaque psoriasis, 227 (75.67%) had only skin lesions, while 73 (24.3%) had concomitant arthritis. Patients with arthritis had a higher mean age (49.98 ± 11.12 versus 46.34 ± 13.21, P = 0.021). The distribution by sex was similar in both groups, as was the age at diagnosis of the disease, which started, on average, at 34 years (34.60 ± 16.10 years in patients with arthritis versus 34.46 ± 15.58 years without arthritis, P = 0.949). A positive family history was significantly more frequent in patients with arthritis (57.5% versus 30.4%, P < 0.001). The disease duration was longer in the group with arthritis (15.63 ± 12.43 versus 11.85 ± 11.12, P < 0.01). More than 80% of the patients in the study had moderate-severe psoriasis, and the PASI values were significantly higher in the group with arthritis (17.08 ± 4.68 versus 12.79 ± 6.75, P < 0.001) (Figure 1). A total of 178 patients (59.4%) with phototype III were evaluated, 105 patients (35 %) with phototype IV, and 17 patients (5.6 %) with phototype V. The serum levels of 25(OH)D were tested more frequently during the summer (164 patients, 54.7%) and to a lesser extent in the winter (23 patients, 7.7%); the two groups did not differ significantly.

PASI was significantly higher in patients with arthritis (P < 0.001).

**Table 1.** Demographic characteristics of patients with psoriasis according to the presence of psoriatic arthritis

Variables	Psoriasis without arthritis n (%)	Psoriasis with arthritis n (%)	P value
	227 (75.67)	73 (24.33)	
**Age, years, (mean ± SD)**	46.34 ± 13.21	49.98 ± 11.12	0.021^a^
**Male/female (n)**	121/106	40/33	-
**Prevalence of men (%)**	53.3	54.8	0.824
**Positive family history [n (%)]**	69 (30.4)	42 (57.5)	0.001^** b^
**Age of diagnosis, in years (mean ± SD)**	34.46 ± 15.58	34.60 ± 16.10	0.949^a^
**Duration of psoriasis, in years (mean ± SD)**	11.85± 11.12	15.63 ± 12.43	0.002^* a^
**PASI (mean ± SD)**	12.79 ± 6.75	17.08 ± 4.68	0.001^** a^
**Severity [n (%)]**			0.001^** b^
Mild	58 (25.5)	1 (1.4)	
Moderate-severe	169 (74.5)	72 (98.6)	
**Fitzpatrick skin phototype [n (%)]**			0.803^b^
III	134 (59.0)	44 (60.3)	
IV	79 (34.9)	26 (35.6)	
V	14 (6.1)	3 (4.1)	
**Season of the year of the test [n (%)]**			0.243^b^
Autumn	40 (17.6)	16 (21.9)	
Winter	15 (6.6)	8 (11.0)	
Spring	48 (21.2)	9 (12.3)	
Summer	124 (54.6)	40 (54.8)	

SD = standard deviation; PASI = Psoriasis Area and Severity Index.

* P < 0.01

** P < 0.001

a Student's t-test

b χ2 Test.

**Figure 2:**
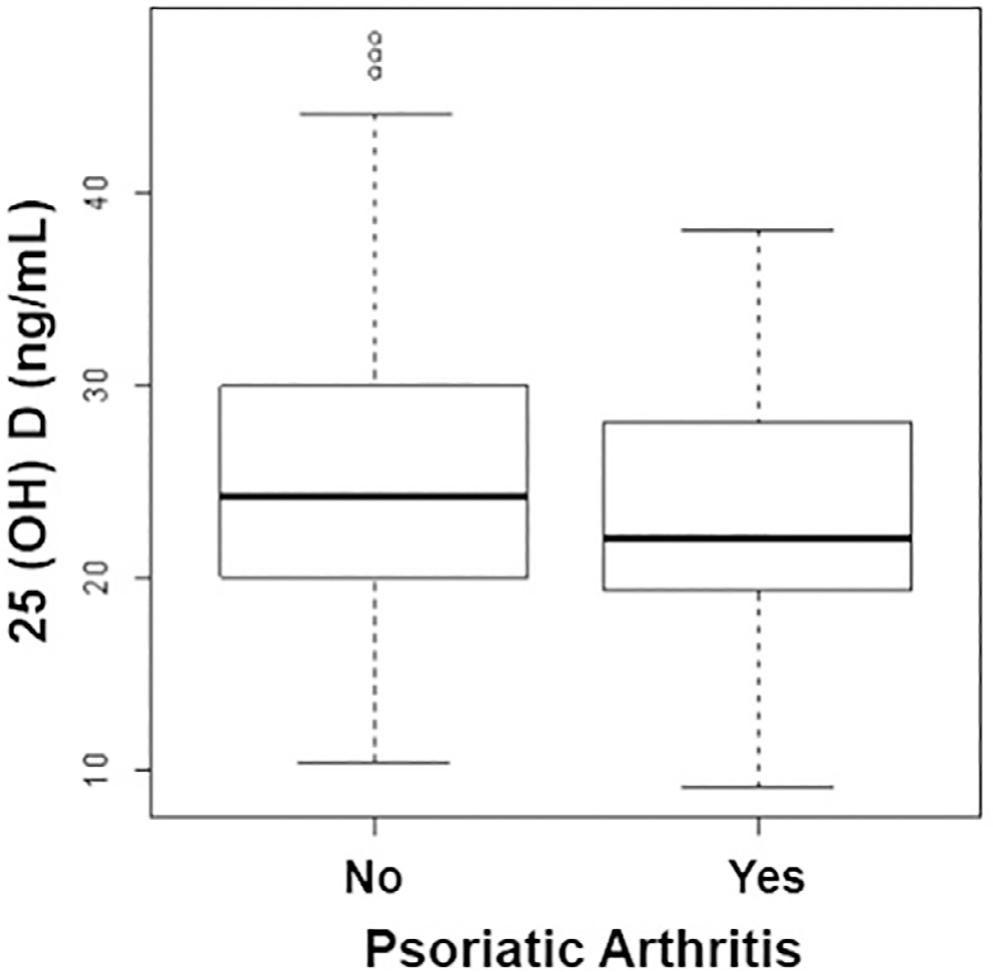
Comparison of serum 25(OH)D levels in psoriasis patients with and without psoriatic arthritis.

Patients with psoriasis and arthritis had significantly lower mean serum 25(OH)D (23.43 ± 6.55 versus 25.39 ± 7.30, P = 0.03) (Figure 2).

Serum 25 (OH) D concentration was significantly lower in patients with arthritis (23.43 ± 6.55 versus 25.39 ± 7.30; P = 0.033).

Vitamin D deficiency (< 20 ng/mL) was present in a greater proportion of patients with arthritis (31.5% versus 23.8%), whereas levels considered sufficient (≥ 30 ng/mL) were proportionally higher in patients with psoriasis without arthritis (25.1% versus 17.8%). The 25(OH)D levels were lower in patients with arthritis in all studied phototypes as well as in all seasons of the year (Table 2).

**Table 2.** Serum 25(OH)D concentration (mean ± SD) in psoriasis patients with and without psoriatic arthritis according to phototype and season of the year

25 (OH) D	Pso without PsA, n (%)	Pso with PsA, n (%)	P value
	227 (75.67)	73 (24.33)	
**Mean ± SD (ng/mL)**	25.39 ± 7.30	23.43 ± 6.55	0.033^a^/0.001^b^
**Minimum**	10.50	9.17	-
**Maximum**	48.0	38.1	-
**25 (OH) D, [n (%)]**			0.301^c^
< 20 ng/mL	54 (23.8)	23 (31.5)	
> 20 and ≤ 30 ng/mL	117 (51.5)	37 (50.7)	
≥ 30 ng/mL	56 (24.7)	13 (17.8)	
**25 (OH) D**			0.265^c^
< 30 ng/mL	171 (75.3)	60 (82.2)	
≥ 30 ng/mL	56 (24.6)	13 (17.8)	
**Skin phototype**			NA
III	25.62 ± 6.67	23.09 ± 5.61	
IV	26.05 ± 8.24	24.71 ± 7.80	
V	19.42 ±4.72	17.43 ± 5.62	
**Season of the year of the test**			NA
Autumn	28.00 ± 7.30	25.48 ± 6.41	
Winter	22.74 ± 6.30	22.53 ± 5.24	
Spring	20.86 ± 6.25	15.44 ± 3.88	
Summer	22.28 ± 5.94	22.03 ± 5.93	

25(OH)D = 25-hydroxyvitamin D; Pso = psoriasis; PsA = psoriatic arthritis; SD = standard deviation

a Student's t-Test;

b One-way analysis of variance post hoc Bonferroni;

c Fisher's Test; NA = not applicable.

The multivariate linear regression model is presented in Table 3 (adjusted model, R2 = 0.31, P < 0.001). The presence of arthritis, severity according to the PASI, family history, age at diagnosis, evolution time, phototype, and season of the year were used as predictor variables, controlling for sex and age.

**Table 3.** Multiple linear regression analysis of independent predictors of serum 25(OH)D concentration in patients with psoriasis

Predictors	β coefficient	P value	95% CI
**Arthritis**			
Absent^*^			
Present	−0.266	0.759	−1.97 – 1.44
**Severity**			
Mild^*^			
Moderate-severe	−6.712	0.000^£^	−8.51 – −4.91
**Family history positive**	0.194	0.797	−1.29 – 1.68
Age at diagnosis	−0.024	0.383	−0.007 – 0.030
Duration of psoriasis	0.006	0.474	−0.06 – 0.08
**Season of the year**			
Winter^£^			
Autumn	3.525	0.017	0.61 – 6.43
Spring	4.512	0.002	1.58 – 7.44
Summer	8.708	0.000^£^	6.07 – 11.33
**Skin phototype**			
III*			
IV	1.556	.035	1.10 a 3.01
V	−3.783	.014	−6.89 a −0.95

* Reference category: 25(OH)D = 25-hydroxyvitamin D; CI = Confidence Interval

£ £P < 0.0001; adjusted R2 = 0.31, P < 0.0001.

The linear regression results demonstrated an inverse and statistically significant correlation between 25(OH)D levels and disease severity. The presence of moderate-to-severe psoriasis was negatively correlated with serum vitamin D levels (β coefficient = –6.712, CI: –8.51, –4.91, P < 0.0001). Serum vitamin D levels were not correlated with the presence of arthritis, positive family history, age at diagnosis, or disease duration. An association between the season of the year in which the serum measurement was performed and the patients’ phototype was evidenced. The positive effect on vitamin D levels, with winter as the reference, increased with the seasons according to the rate of ultraviolet radiation: spring (β Coefficient = 4.512, confidence interval, CI: 1.58 to 7.44,P = 0.002) and summer (β Coefficient = 8.708, CI: 6.07 to 11.33, P < 0.0001). There was an inverse association between vitamin D levels and the highest phototypes, such as Phototype V (β Coefficient = –3.783, CI: –6.89 to –0.95, P = 0.014) and phototype IV (β Coefficient = 1.56, CI:1.10 to 3.01, P = 0.035).

The 25(OH)D levels were inversely correlated with PASI values (patients without arthritis Pearson's r = –0.59, P < 0.001 and with arthritis r = –0.52, P < 0.001).

To confirm the inverse correlation between vitamin D levels and PASI scores, we developed a binary logistic regression model (Table 4). This model confirmed a strong association between PASI and vitamin D deficiency (< 30 ng/mL) (odds ratio, OR 1.78, CI: –0.20 to –0.53, P < 0.001).

**Table 4.** Binary logistic regression model for 25(OH)D deficiency in psoriatic patients

Variable	β Coefficient	OR	CI 95%	P value
Arthritis	0.513	1.67	0.70 – 3.89	0.23
Duration of disease	0.023	1.02	0.99 – 1.05	0.16
Family history positive	0.601	1.82	0.92 – 3.64	0.08
Age at diagnosis	−0.062	0.97	0.96 – 1.01	0.62
PASI	−0.240	1.78	−0.20 – 0.53	0.001^*^

25(OH)D, 25-hydroxyvitamin D; OR = Odds Ratio; CI = Confidence Interval; PASI = Psoriasis Area and Severity Index.

* P < 0.0001.

## DISCUSSION

Hypovitaminosis D (< 30 ng/mL) was highly prevalent in patients with psoriasis with and without PsA (82.2% and 74.9%, respectively). An inverse correlation of PASI with vitamin D was found (without PsA r = –0.59 and with PsA r = –0.52, P < 0.001), and multivariate regression revealed that hypovitaminosis D was associated with the severity of the disease, the season and phototype. An inverse association between PASI and the serum level of 25(OH)D was confirmed by binary logistic regression between PASI and vitamin D deficiency (< 30 ng/mL) (odds ratio, OR 1.78 CI: –0.20–0.53, P < 0.001).

Some previous studies have confirmed the association between vitamin D deficiency and psoriasis^9,10,14,15,16,17,18^; however, in contrast to those studies, a few studies have shown no correlation between them.^19–21^ There have been a few studies investigating the comparison between psoriatic patients with and without arthritis.^18,22,23,24^ In situations of low concentrations of 25(OH)D, the immune system favors the development of self-reactive T cells directed against the body's own tissues, and the synthesis of pro-inflammatory cytokines (IL-12 and IFN-γ), predisposing the body to an increased risk of developing autoimmune diseases such as diabetes, rheumatoid arthritis, multiple sclerosis, and inflammatory bowel diseases.^25–27^ In psoriasis, the immune system behaves similarly under low concentrations of 25(OH)D and in addition, 25(OH)D is believed to inhibit the production of Th1 and Th17 inflammatory cytokines.^28^

Orgaz-Molina et al.^10^ evaluated 43 white patients with plaque psoriasis, 7% of which were associated with psoriatic arthritis with a mean PASI of 4.42, and observed a strong association between psoriasis and vitamin D insufficiency according to logistic regression (< 30 ng/mL) (OR 2.89 CI 95 1.02 to 7.64). However, vitamin D deficiency was not associated with PASI, neither with disease duration nor with the presence of arthritis, which finding differs from our results.^10^

A lack of correlation of vitamin D insufficiency with PASI despite a high prevalence of deficiency in psoriatic patients, was reported in a study that evaluated 43 patients with psoriasis, 55 with rheumatoid arthritis (RA), and 40 healthy controls; serum levels were significantly lower in patients with psoriasis and RA (P < 0.001).^18^

Our findings were consistent with those of other studies^14,15,17^ which demonstrated a higher level of 25(OH)D deficiency in patients with psoriasis and 25(OH)D deficiency was negatively correlated with PASI.

Studies in the southern hemisphere are rare. In places with latitudes above 37°N or below 35°S there is a decrease in the incidence of ultraviolet B radiation during the winter months, and the chances of vitamin D deficiency increase.^27,28^

Zuchi et al.^19^ reported a study in Curitiba, South Brazil (250S 490W), which compared serum vitamin D levels in 20 psoriasis patients and 20 control patients. Of the 20 psoriasis patients, 15 had plaque psoriasis and 5 had palmoplantar psoriasis. However, the patients studied had low mean PASI (2.4 ± 3.6). There were no differences between the two groups, and the authors reported lower serum vitamin D levels in women than men (20.85 ± 6.70 versus 25.35 ± 2.90; P = 0.031).^19^ These data disagree with our study results. This can be explained in part by the fact that our study was conducted in a place located at latitude 210S 430W, which theoretically would be related to adequate levels of vitamin D, and they studied other clinical forms of psoriasis; the phototypes were lower (I and II). It is important to point out that patients with severe psoriasis, with extensive areas of involvement, tend to cover themselves to hide their lesions, which would consequently explain the lower sun exposure and production of vitamin D. 

Regarding the comparison between psoriatic patients with and without arthritis: Orgaz-Molina et al.^18^ compared 61 patients with psoriatic arthritis and 61 patients without psoriatic arthritis, found no correlation with disease severity, and concluded that 25(OH)D was inversely related to metabolic parameters in patients with psoriasis without arthritis.^18^ However, the authors selected patients with mild disease (PASI = 4.76 ± 5.31 in the group without arthritis versus 3.66 ± 3.48 in the group with arthritis).

On the other hand, Kincse et al.^22^ found a prevalence of hypovitaminosis D (< 30 ng/mL) in 63% of cases, with inverse correlations between serum vitamin D levels and psoriasis severity (PASI), and arthritis activity.^20^ The influence of season and latitude on serum vitamin D levels was investigated by Touma et al.^24^ who studied 302 patients with psoriatic arthritis, 201 in Toronto (43° 40’ N) and 102 in Israel (32° 46’ N), in summer and winter. It was found that levels < 75 nmol/L (30 ng/mL) were 58.7% versus 57.9% in winter in Toronto versus Israel, respectively, and 58.6% versus 64.9% in summer. The authors also evaluated the effect of skin phototype and season, concluding that there were no differences in these variables in each studied group or between the two groups. They also did not find an association between PASI and arthritis activity marker levels. However, the selected individuals had lower PASI averages calculated by rheumatologists (3.59 ± 5.09 in winter and 3.44 ± 5.59 in summer).

In this context, there are reports of inadequate levels of vitamin D, even in individuals with adequate exposure to the sun, due to other related factors, such as high altitudes, obesity, and skin pigmentation.^26–29^

The limitations of our study include the absence of a dietary and sun exposure survey (with time and duration of exposure), and the fact that phototypes I, II, and VI were not represented.

## CONCLUSIONS

In conclusion, we emphasize that hypovitaminosis D is highly prevalent in psoriatic patients with and without psoriatic arthritis and in patients with plaque psoriasis with or without arthritis. Furthermore, there was an inverse correlation between 25(OH)D levels and disease severity (PASI). Finally, there were associations between 25(OH)D levels and season of the year and skin phototype. These findings highlight the importance of vitamin D status in these patients and emphasize the need for its regular monitoring in addition to considering vitamin D supplementation, especially in patients with moderate to severe psoriasis, with high phototypes, and during autumn and winter months.
